# Work Stress, Overcommitment and Somatization among Early Childhood Professionals during COVID-19: A Cross-sectional Study

**DOI:** 10.1192/j.eurpsy.2023.974

**Published:** 2023-07-19

**Authors:** S. Gritzka, P. Angerer, M. Diebig

**Affiliations:** Institut für Arbeits-, Sozial- und Umweltmedizin, Heinrich-Heine-Universität Düsseldorf, Düsseldorf, Germany

## Abstract

**Introduction:**

In addition to posing major public health challenges, the COVID-19 pandemic has affected psychological and physical stressors in workplaces as well as strain by workers worldwide. Even prior COVID-19, the working conditions of early childhood professionals (ECPs) were described as critical leading to (psycho)somatic complaints. Despite the high societal relevance of ECPs and the potential increase in stressors caused by COVID-19, there is a lack of empirical evidence to which extent work-related demands had an adverse effect on ECPs health during COVID-19.

**Objectives:**

We aimed to obtain updated prevalence rates of somatic symptoms of ECPs as well as unfavourable working conditions inducing stress (in terms of an effort-reward imbalance; ERI) in childcare. The overall objective was to establish associations between work stress and health during the early phase of the pandemic. Given the tendency of ECPs to overcommit themselves, we further explored the potential moderating role of overcommitment.

**Methods:**

Between June 2020 and May 2021, questionnaire data was collected using validated instruments (i.e., ERI, PHQ-15) from ECPs in childcare centers as well as from family providers (*N* = 1.009). ECPs were informed about the study and contacted through the youth welfare office. Multiple logistic regression tested the influence of ERI and overcommitment on the severity of somatic symptoms. It was controlled for age, gender as well as leadership position. SPSS 28.0 was used to analyze the data.

**Results:**

The ERI ratio of the sample averaged 1.28 (*SD* = 0.45), with 72.7% of subjects exceeding the critical cut-off value > 1, indicating a gratification crisis. The averaged sum score of the PHQ-15 was 8.99 (*SD* = 5.43). Based on a PHQ-15 cut-off ≥ 10, the overall prevalence of somatization at a moderate to high level is estimated to be 44.6%. The mean overcommitment score was 15.14 (*SD* = 3.55) and 23.4% of ECPs were in the highest overcommitment tertile. ERI (*OR* = 4.12, 95% *CI*: 2.73 - 6.22) and overcommitment (*OR* = 5.20, 95% *CI*: 2.17 - 12.47) were associated with an increased likelihood of greater severity of somatic symptoms. Yet, the interaction effect between both predictors remained non-signifcant. Being female and having no leadership position were predictors for a moderate to high level of somatization.

**Conclusions:**

The results demonstrate the high relevance of work stress for somatic health among ECPs in the midst of a pandemic. Given the high overall prevalence of somatic symptoms and the female-dominated work sector, ECPs may be at high risk for somatoform disorders. There remains a strong need for action to reduce work-related stress in order to decrease the somatic symptom burden. Large-scale and longitudinal studies are needed to further investigate coping with and persistence of somatic symptoms among this occupational group.

**Disclosure of Interest:**

None Declared

